# Clinical, Histological, and Immunohistochemical Insights into a Canine Hepatic Myofibroblastic Sarcoma

**DOI:** 10.3390/vetsci12060521

**Published:** 2025-05-26

**Authors:** Valentina Rinaldi, Laura Nordio, Massimo Vignoli, Stefano Masci, Lorenzo Ressel, Paolo Emidio Crisi

**Affiliations:** 1 Department of Veterinary Medicine, University of Teramo, Località Piano D’Accio, 64100 Teramo, Italy; mvignoli@unite.it (M.V.); pecrisi@unite.it (P.E.C.); 2 San Marco Veterinary Clinic and Laboratory, Division of Pathology, 35030 Veggiano, Italy; laura.nordio.90@gmail.com; 3 Clinica Veterinaria Colli Innamorati, Via Colli Innamorati 21, 65125 Pescara, Italy; stefanomasci@hotmail.it; 4 Department of Veterinary Anatomy Physiology and Pathology, Institute of Infection, Veterinary and Ecological Science, Faculty of Health and Life Science, University of Liverpool, Chester High Road, Neston CH64 7TE, UK; ressel@liverpool.ac.uk

**Keywords:** dog, tumor, liver, sarcoma

## Abstract

Hepatic sarcomas are rare and aggressive tumors in veterinary medicine, with limited reports in the literature. This case report describes a canine hepatic myofibroblastic sarcoma in a 5-year-old spayed female Dobermann. The dog presented with abdominal enlargement and was diagnosed with a large hepatic mass following comprehensive diagnostic evaluations, including blood tests, imaging, and histopathology. Histological and immunohistochemical analyses confirmed the tumor’s myofibroblastic origin, characterized by positivity for markers such as vimentin, glial fibrillary acidic protein (GFAP), nerve growth factor receptor (NGFR), alpha-smooth muscle actin (SMA), and muscular actin (HHF35). Treatment involved a combination of intense-dose chemotherapy using doxorubicin and a subsequent metronomic chemotherapy protocol, which resulted in prolonged survival of over 690 days at the time this manuscript was written. This case highlights the importance of extensive diagnostic and immunohistochemical profiling in the accurate classification of and treatment planning for hepatic sarcomas, and emphasizes the role of advanced veterinary diagnostics in improving patient outcomes. Future studies with larger sample sizes are needed to enhance understanding of the biological behavior and optimal therapeutic strategies for such rare tumors.

## Simple Summary

Hepatic sarcomas are rare and aggressive tumors in veterinary medicine. This case report describes a hepatic myofibroblastic sarcoma in a 5-year-old spayed female Dobermann presenting with abdominal enlargement and a large liver mass. The diagnosis was confirmed through histopathology and immunohistochemistry that revealed a myofibroblastic origin with positive markers including vimentin, glial fibrillary acidic protein (GFAP), nerve growth factor receptor (NGFR), alpha-smooth muscle actin (SMA), and muscular actin (HHF35). The dog was treated with doxorubicin-based intensive chemotherapy followed by a metronomic protocol, and achieved a survival time exceeding 690 days. This case underscores the value of comprehensive diagnostic approaches and highlights the need for further research to guide treatment of these rare tumors.

## 1. Introduction

Mesenchymal tumors of the liver are rare in both humans and animals. Hemangiosarcoma is the most commonly reported type, occurring both as primary and metastatic hepatic tumors [1,2,3]. Other types of hepatic mesenchymal neoplasms in dogs include leiomyosarcomas and fibrosarcomas [3,4,5], while osteosarcomas and mesenchymomas are less frequently observed [6,7]. In 2013, Park and colleagues provided a histological characterization of a hepatic malignant peripheral nerve sheath tumor (MPNST) in a dog [8]; only 11 cases of this same hepatic tumor have been reported in humans [9]. Generally, hepatic sarcomas demonstrate aggressive biological behavior and high metastatic potential, leading to poor prognoses. Affected dogs are often euthanized upon diagnosis due to the severity of the condition [4,8]. This case report presents a canine hepatic myofibroblastic sarcoma, detailing its clinical progression and associated outcome.

## 2. Case History

A 5-year-old spayed female Dobermann (32 kg bodyweight) was referred to the Veterinary Teaching Hospital (VTH) of the University of Teramo for an oncological consultation. One month earlier, the dog was presented to the referring colleagues due to abdominal enlargement. The patient underwent blood tests (hematologic and biochemical) and basic staging, with thoracic radiographs in three views and an abdominal ultrasound (US). Blood tests revealed a moderate regenerative anemia and a marked elevation in liver damage enzymes. All hematological and biochemical data are presented in Table 1.

**Figure 1:**
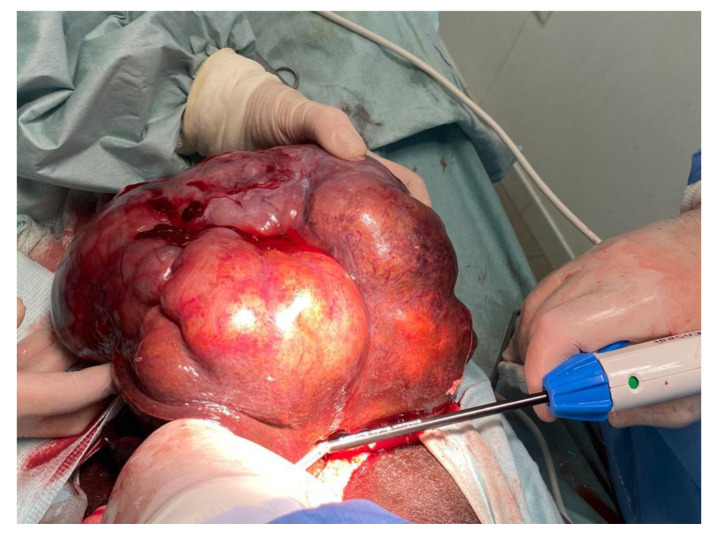
Macroscopic presentation of the tumor.

The US revealed a mass with a maximum diameter of approximately 20 cm affecting the quadrate and caudate hepatic lobes and abdominal effusion. Subsequently, the dog underwent surgery for the removal of the mass (Figure 1), and the tissue was placed in 10% buffered formalin for histopathological examination. Formalin-fixed and paraffin-embedded samples (FFPEs) were sectioned and routinely stained with haematoxylin and eosin (H&E).

**Figure 2:**
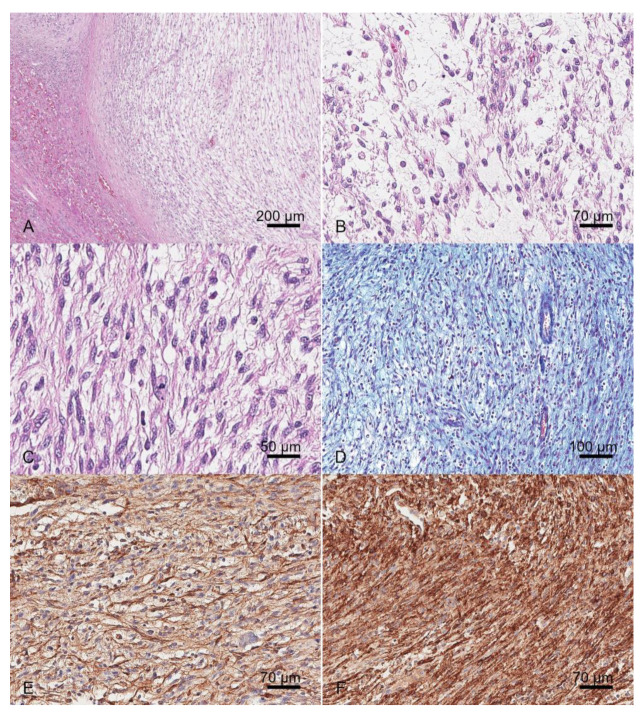
Sarcoma, liver, dog. (**A**–**C**). Hematoxylin and eosin (H&E), 5× (**A**), 20× (**B**), and 30× (**C**). Neoplasm characterized by short bundles, embedded in myxoid matrix of spindle-to-oval-to-round, occasionally vacuolated, cells. (**D**). Alcian blue stain, 10×. Myxoid matrix stained blue with Alcian blue stain. (**E**). Immunohistochemistry for muscular actin (HHF35), 20×. Neoplastic cells were positive for HHF35. (**F**). Immunohistochemistry for nerve growth factor receptor (NGFR), 20×. Neoplastic cells were positive for NGFR.

On histopathological evaluation, the hepatic parenchyma was focally effaced by a nodular, infiltrative, sparsely cellular, moderately demarcated, unencapsulated neoplasm. Neoplastic cells were organized in short bundles embedded in variable amounts of myxoid matrices that tested positive with Alcian blue stain (BioOptica, Milan, Italy) (Figure 2). Neoplastic cells were spindle-to-oval-to-round and 8–12 microns in transverse diameter, with a low-to-intermediate N/C ratio, indistinct cell borders, and moderate or abundant homogeneous eosinophilic, or occasionally vacuolised, cytoplasm. Nuclei were oval and paracentric, with granular chromatin and 1–2 small nucleoli. Anisocytosis and anisokaryosis were moderate and the mitoses range was 0–5 per HPF (mitotic count: 22 total mitoses in 10 HPFs equal to 2.37 mm^2^).

The adjacent parenchyma was characterized by moderate hepatocellular hydropic degeneration and scattered lipogranulomas. A diagnosis of sarcoma with myxoid differentiation was made. The phenotype was further characterized by immunohistochemistry. A panel of immunohistochemical stains was carried out with antibodies antivimentin, pan-cytokeratins (CKAE1-AE3), cytokeratin-7 (CK7), alpha-smooth muscle actin (SMA), muscular actin (HHF35), desmin, S100, glial fibrillary acidic protein (GFAP), nerve growth factor receptor (NGFR), and CD117, as detailed in Table 2. Immunohistochemistry was performed with an automatic immunostainer (Bond RX, Leica, Wetzlar, Germany).

Neoplastic cells were diffusely positive for vimentin. About 70% of neoplastic cells were positive for GFAP and NGFR, while about 50% were positive for SMA and HHF35 (Figure 2). Immunohistochemistry for CKAE1-AE3, CK7, S100, CD117, and desmin had negative results.

A final diagnosis of myofibroblastic sarcoma was made. One month after surgery, the dog was referred for an oncologic consultation. No abnormal clinical findings were observed, and both complete blood count and serum biochemistry revealed values within reference intervals. Staging was performed using total-body computed tomography (CT) with 1.3 mm slice thickness. The CT study revealed lung nodules up to 4 mm, with moderate bilateral sternal lymphadenomegaly (15 mm dorsoventral diameter), both homogenous and moderately contrast-enhanced. No pathological findings were detected in the liver area on CT.

A dose-intense chemotherapy protocol was started with doxorubicin at 30 mg/m^2^ intravenously for four cycles every 21 days. No adverse effects were recorded according to criteria published by the Veterinary Cooperative Oncology Group (VCOG-CTCAE) [10]. As a supportive therapy, maropitant (2 mg/Kg PO q24 h) was administered for three days from the start of chemotherapy.

The dog underwent a CT scan for end-staging, which revealed stable disease according to RECIST criteria [11]. A mild enlargement of the lung nodule in the right cranial lung lobe, measuring up to 6 mm in diameter, was noted. The right sternal lymph node decreased from 15 to 10 mm in dorsoventral diameter, while the left one measured 6 mm. Both sternal lymph nodes appeared homogenous; however, the right lymph node exhibited stronger contrast enhancement (30 HU pre-contrast and 135 HU post-contrast) compared to the left (30 HU pre-contrast and 100 HU post-contrast).

A metronomic chemotherapy (MC) protocol with cyclophosphamide (10 mg/m^2^ PO q48 h), piroxicam (0.3 mg/Kg PO q48 h), and thalidomide (2 mg/Kg PO q24 h) was started. A diagnostic follow-up, including thoracic radiography in three views and abdominal US, was planned every 3 months.

Disease progression was evaluated based on an increase in the lung nodule, with a maximum diameter > 30% compared to the previous CT scan with a progression-free interval (PFI) of 153 days. At the time of manuscript preparation, no pathological findings were observed in the abdominal organs or other regions of the liver on US. The dog was alive at the time of writing, and therefore survived over 690 days after the diagnosis.

## 3. Discussion

Hepatic sarcomas are very rare tumors, and, in general, reports of abdominal sarcomas are limited [12,13]. Therefore, the use of chemotherapy is also restricted. Immunohistochemical findings confirmed the diagnosis of sarcoma, excluding a myxoid liposarcoma and a gastrointestinal stromal tumor (GIST) as possible differential diagnoses. Specifically, immunolabelling for SMA, HHF35, GFAP, and NGFR was suggestive of a possible myofibroblastic origin of the neoplastic cells, primarily consistent with a myofibroblastic sarcoma.

Similar tumors have been described by Filips and colleagues [14] in humans. These neoplastic conditions, classified as inflammatory myofibroblastic tumors (IMTLs) of the liver, are characterized by certain features, including a combination of myxoid/vascular, spindle cell, and hypocellular fibrous patterns that overlap with those observed in this case, associated with a strong inflammatory infiltrate that may include lymphocytes, plasma cells, and occasionally neutrophils. However, in humans, IMTLs often express cytoplasmic ALK (in approximately half of cases); this marker could not be tested in this case. These findings suggest possible molecular differences between the two entities. However, given the differences in the diagnostic panel used, a direct comparison remains speculative. Moreover, a further significant morphological difference is that no inflammation was observed in association with the neoplastic proliferation of this tumor. The absence of plasma cells and of a prominent inflammatory infiltrate is considered a useful finding to discriminate other types of sarcomas from IMT(Gleason 2008) [15].

Another differential diagnosis included an Ito cell tumor. Activated hepatic fibroblasts with myofibroblastic characteristics play a role in the pathogenesis of hepatic fibrosis. These cells include subpopulations such as portal myofibroblasts and perisinusoidal hepatic stellate cells (HSCs, also termed Ito cells) [16]. HSCs reside in the space of Disse and are able to produce an extra-cellular matrix and transform, upon activation, into a myofibroblastic phenotype, contributing to collagen deposition [17]. Tumors originating from Ito cells are rarely reported in mice and rats [18,19]. These tumors consist of nodular aggregates of round-to-spindle cells, often containing clear vacuoles, identified as lipid droplets by oil red O stain, and exhibit immunoreactivity for vimentin, actin, and desmin [19]. Normal portal myofibroblasts and HSCs immunolabel for GFAP in rats and humans, but in contrast are GFAP-negative in dogs [16,20]. In the normal canine liver, portal myofibroblasts and HSCs can be easily identified by immunohistochemical staining for both SMA and HHF35. Portal myofibroblasts stain positively for vimentin, while HSCs are typically vimentin-negative; both cell types, however, are GFAP-negative [16], which contrasts with the phenotype observed in this case, thus not supporting the diagnosis of an Ito cell tumor. Therefore, we favor the hypothesis of a myofibroblastic sarcoma of unspecified origin. Lastly, an MPNST should also be considered as a differential diagnosis for the present case. The immunoreactivity for muscular markers, such as SMA, in NSTs is generally considered atypical [21]; however, it has rarely been reported. Neural crest-derived tumors, such as those originating from Schwann cells, could show expression of SMA on rare occasions, probably as a divergent differentiation of some Schwann cells towards smooth muscle or myofibroblasts [22,23].

Intensive dose chemotherapy in hepatic sarcomas has been investigated in cases of hemangiosarcoma using a doxorubicin-based protocol [24]. A previous study focused on the use of doxorubicin in grade III soft tissue sarcomas (STSs) and showed no survival difference between treated and untreated dogs, even if, in the 39 cases included, only 8 were visceral, and none were hepatic [25]. In humans, STSs are considered rare and consist of various subtypes; therefore, extensive data to establish a therapeutic gold standard are lacking. Immunotherapy is certainly the most innovative treatment; however, a multimodal approach remains common, combining surgery, radiotherapy, and the use of doxorubicin with or without ifosfamide [26,27]. In canine tumors, MC has been described as effective in preventing the recurrence of these incompletely resected STSs, allowing longer disease-free times compared to surgery alone [28]. A limitation of this case report is the lack of histological characterization of the multiple pulmonary lesions, which were considered metastatic based only on imaging findings. However, it was deemed inappropriate to pursue the risks associated with an invasive diagnostic procedure targeting 4 mm pulmonary nodules in a patient already diagnosed with a hepatic tumor of 20 cm. Moreover, the progression of these lesions in an adult dog was more suggestive of neoplastic behavior than of an inflammatory process.

## 4. Conclusions

This case report describes a rare case of sarcoma in a dog’s liver, classified, based on morphology and phenotype, as a myofibroblastic sarcoma. This case enriches data in the scientific literature regarding liver sarcomas, which are rare in both animals and humans. The use of an extensive immunohistochemical panel allowed for a definitive diagnosis and a correct classification of a previously unreported liver tumor, highlighting the importance of advanced diagnostics in pathology. Sarcomas, whether cutaneous or visceral, are rarely fully characterized in clinical practice, which limits accurate assessment of biological behavior and subsequent clinical decisions and prognosis. A more precise diagnosis enables clinicians to select appropriate post-surgical treatments, which, as demonstrated in this case report, may enhance the expected survival of oncology patients. On the other hand, the authors cannot conclusively attribute survival to the specific treatment used, further underscoring that a more accurate characterization of sarcomas could pave the way for more precise prognostic evaluations.

Diagnostics in the future will probably move towards a molecular classification of neoplasms that incorporate next generation technologies, leading to a targeted medical approach tailored to the individual needs of each patient. In human medicine, many soft tissue neoplasms have characteristic genetic abnormalities that have already led to a complex classification system for many soft tissue tumors, which incorporate histopathological characteristics, molecular genetic features, and potential molecular grading [29,30]. Human sarcoma are classified into more than 50 different subtypes with different biological behaviors [30]. Genetic changes can assist the pathologist in the differential diagnosis of some of these entities and guide the clinician in targeted therapies, with modern clinical trials that focus on specific sarcoma subtypes [29,31].

Future scientific studies with adequate case numbers and comprehensive immunohistochemical and genetic analyses are essential for better understanding the biological behavior of myofibroblastic sarcomas in dogs.

## Figures and Tables

**Table 1:** Hematological and serum biochemical data.

Parameters	Value	Range
Hemoglobin	6.3	12–18 g/dL
Hematocrit	18.4	37.0–55.0%
Red Blood Cells	2,640,000	5,500,000–8,500,000/mm^3^
Platelets	145,000	160,000–500,000/mm^3^
White Blood Cells	12,950	6000–17,000/mm^3^
Mean Corpuscular Volume	69.6	60.0–77.0 fL
Mean Platelet Volume	31.6	6.6–10.9 fL
Mean Corpuscular Hemoglobin	34	32.0–38.0 g/dL
Mean Corpuscular Hemoglobin Concentration	23.7	19.5–24.5 pgr
Red Cell Distribution Width	28.8	13.0–15.7%
Lymphocytes	4278	1000–4800/mm^3^
Monocytes	924	100–1400/mm^3^
Neutrophils	7003	3000–12,000/mm^3^
Eosinophils	100	0–750/mm^3^
Basophils	12	0–180/mm^3^
Reticulocytes	14,410	0–60,000/mm^3^
Reticulocyte Percentage	5.45%	0–1.5%
Corrected Reticulocyte Percentage	2.20%	0–1%
Glucose	100 mg/dL	70–125
Urea	50 mg/dL	18–55
Creatinine	1.1 mg/dL	0.65–1.35
Total Bilirubin	0.2 mg/dL	0.07–0.34
AST	356 U/L	20–42
ALT	574 U/L	20–55
DGGR Lipase	60 U/L	10–130
GGT	5 U/L	0–5.8
CK	300 U/L	50–290
Calcium	10.5 mg/dL	9.0–11.8
Corrected Calcium	10.4 mg/dL	9.0–11.8
Phosphorus	3.9 mg/dL	2.6–4.9
Total Proteins	6.6 mg/dL	5.3–7.9
Cholesterol	300 mg/dL	140–350
Triglycerides	230 mg/dL	30–120
Albumin	3.6 g/dL	2.5–3.7
Sodium	144 mEq/L	143–154
Potassium	4.1 mEq/L	3.9–5.3
Chloride	110 mEq/L	108–118
Corrected Chloride	112 mEq/L	108–118
ALP	1500 U/L	42–180
Iron	248 μg/dL	72–168
UIBC	201 μg/dL	140–296
C-Reactive Protein	5.2 mg/dL	0.0–1
Na/K Ratio	35	
Globulins	3 g/dL	2.5–4.5
A/G Ratio	1.2	0.6–1.3
TIBC	449 μg/dL	268–404

AST: Aspartate Aminotransferase; ALT: Alanine Aminotransferase; CK: Creatinine Kinase; DGGR: 1,2-o-dilauryl-rac-glycero-3-glutaric acid-(6′-methylresorufin; GGT: Gamma-Glutamyl Transferase; ALP: Alkaline Phosphatase; UIBC: Unsaturated Iron-Binding Capacity; Na/K Ratio: Sodium/Potassium Ratio; TIBC: Total Iron-Binding Capacity; A/G Ratio: Albumin/Globulin Ratio.

**Table 2:** Details of immunohistochemical protocols.

Antigen	Dilutions	Source	Epitope Retrieval	Positive Control
Vimentin	1:1000	Agilent Dako; Santa Clara, CA, United States	Heat-induced epitope retrieval, pH 6, 10′	Vascular tunica media
Pan-cytokeratins (CKAE1-AE3)	1:1000	Agilent Dako; Santa Clara, CA, United States	Enzymatic digestion, 10′	Bile duct
Cytokeratin-7 (CK7)	1:200	Agilent Dako; Santa Clara, CA, United States	Heat-induced epitope retrieval, pH 6, 15′ at 97 °C	Bile duct
Smooth muscle actin (SMA)	1:2000	Agilent Dako; Santa Clara, CA, United States	None	Vascular tunica media
Muscular actin (HHF35)	1:500	Agilent Dako; Santa Clara, CA, United States	Heat-induced epitope retrieval, pH 6, 10′ at 97 °C	Vascular tunica media
Desmin	1:200	D33; Histo-line, Pantigliate, Itay	Heat-induced epitope retrieval, pH 6, 20′ at 97 °C	Vascular tunica media
S100	1:100	Leica; Wetzlar, Germany	None	Nerve
Glial fibrillary acidic protein (GFAP)	1:3000	Agilent Dako; Santa Clara, CA, United States	Heat-induced epitope retrieval, pH 6, 20′	Nerve
Nerve growth factor receptor (NGFR)	1:4000	Invitrogen, Thermo Fisher; Waltham, MA, United States	Heat-induced epitope retrieval, pH 6, 20′	Nerve
CD117	1:500	Agilent Dako; Santa Clara, CA, United States	Heat-induced epitope retrieval, pH 9, 20′	Mast cell tumor

## Data Availability

Raw data can be made available upon reasonable request.
